# Coaxial Electrospun Porous Core–Shell Nanofibrous Membranes for Photodegradation of Organic Dyes

**DOI:** 10.3390/polym16060754

**Published:** 2024-03-09

**Authors:** Yaoyao Yang, Shengwei Zhou, Xianyang Cao, He Lv, Zhiyuan Liang, Rui Zhang, Fujia Ye, Dengguang Yu

**Affiliations:** School of Materials and Chemistry, University of Shanghai for Science and Technology, 516 Jungong Road, Shanghai 200093, China; 213353182@st.usst.edu.cn (S.Z.); 202342936@st.usst.edu.cn (X.C.); 201850158@st.usst.edu.cn (H.L.); 212203084@st.usst.edu.cn (Z.L.); 212203081@st.usst.edu.cn (R.Z.); 1935041028@st.usst.edu.cn (F.Y.)

**Keywords:** electrospinning, core–shell nanofibers, porous membranes, AgCl/ZnO heterojunction, photodegradation

## Abstract

In this study, a series of AgCl/ZnO-loaded nanofibrous membranes were prepared using coaxial electrospinning. Their physical and chemical characteristics were evaluated by SEM, TEM, XRD, XPS, IR, PL, and UV–visible spectrometer, and the photocatalytic experiments using methylene blue (MB) as a model pollutant. The formation of AgCl/ZnO heterojunction and the structure of core–shell nanofibers with porous shell layer were confirmed. AgCl/ZnO photocatalysts were also effectively loaded onto the surfaces of the porous core–shell nanofibers. The results of photocatalytic experiments revealed that the AgCl/ZnO (M_AgCl_:M_ZnO_ = 5:5)-loaded nanofibrous membrane achieved a degradation efficiency of 98% in just 70 min and maintained a photocatalytic efficiency exceeding 95% over the first five experimental cycles, which successfully addressed the issues of photocatalytic efficiency loss during the photodegradation of MB with AgCl/ZnO nanoparticles as photocatalyst. The photodegradation mechanism was also researched and proposed.

## 1. Introduction

Due to the swift advancement of contemporary society, organic pollutants [1,2,3] like dyes, pesticides, and fertilizers have emerged as the principal factors contributing to water pollution. The rapid and efficient removal of water pollutants has become a popular topic and a focus of research in the environmental field. Organic dyes, as common pollutants, can lead to reduced oxygen levels, increased toxicity, and excessive nutrient contents in water bodies. Photocatalysis induces oxidation–reduction reactions in semiconductor materials [4,5,6,7,8,9] to degrade organic pollutants and is now a highly regarded green and sustainable development strategy. Heterostructures formed by two or more photocatalytic materials often exhibit a wider range of light absorption wavelengths compared with single photocatalysts [10,11,12,13]. By effectively suppressing the recombination of electron–hole pairs, photocatalytic performance can be enhanced. 

Photocatalytic nanoparticles are often in the form of a powder, which has disadvantages, such as easy aggregation and difficult recovery after a reaction, resulting in a loss of photocatalytic activity and decreased recyclability [14,15]. Photocatalytic nanoparticles can be loaded on nanofiber skeletons by combining electrospinning technology with photocatalysis [16,17], which can effectively improve recycling performance while ensuring high catalytic efficiency. In recent years, the rapid advancement of multifluid electrospinning technology [18,19] has resulted in a diverse range of internal structural designs for electrospun nanofibers. Additionally, the controllable manufacturing of multilevel complex structures [20,21] has significantly enhanced the functionality of fiber materials.

Porous nanofibers have high specific surface areas, making them ideal carriers for photocatalysts [22,23,24,25,26]. However, such porous structures may reduce the mechanical properties of fibers. Nanofibers that are prepared using coaxial electrospinning technology, featuring both a core–sheath structure and a porous sheath layer, exhibit outstanding mechanical properties and a high specific surface area, effectively overcoming the limitations of conventional porous nanofibers.

In this study, silver chloride (AgCl) nanoparticles (NPs) were prepared using a water bath method and then combined with zinc oxide (ZnO) to form AgCl/ZnO composite photocatalytic particles. Porous nanofibers were prepared using cellulose acetate (CA) and polycaprolactone (PCL) as polymer matrices, and AgCl/ZnO was loaded onto their surfaces. A series of tests were carried out on the prepared functional fiber membranes to examine their surface morphology and physical properties. The performance and mechanism of the photocatalytic degradation of methylene blue by porous nanofiber membranes loaded with photocatalysts were investigated through transient photocurrent response, electrochemical impedance, free radical scavenging, and cyclic degradation experiments.

## 2. Materials and Methods

### 2.1. Reagents

The materials used in this study were a silver nitrate standard solution (0.1000 mol/L, Macklin), zinc oxide nanoparticles (30 ± 10 nm, Shanghai Macklin Biochemical Technology Co., Ltd., Shanghai, China), hydrochloric acid (HCl, 36–38%, analytical-grade, China Pharmaceutical, Shanghai, China), polyvinylpyrrolidone (PVP K10, China Pharmaceutical), anhydrous ethanol (EtOH, 99.7%, analytical-grade, Macklin, Shanghai, China), deionized water (H_2_O), methylene blue (MB, >98%, Macklin), cellulose acetate (CA, 39.3–40.3 wt.% acetyl, Sigma-Aldrich, St. Louis, MO, USA), polycaprolactone (PCL, 99%, Mw = 80,000, Sigma-Aldrich), hexafluoroisopropanol (HFIP, 99.5%, analytical-grade, Mw = 168.04, Macklin), trichloromethane (CF, ≥99.0%, China Pharmaceutical), ethylenediaminetetraacetic acid disodium salt (Na_2_-EDTA, 99.96%, Macklin), isopropanol (IPA, ≥99.9%, Macklin), and benzoquinone (P-BQ, ≥99.5%, Macklin). These regents were used directly after purchase.

### 2.2. Sample Preparation

#### 2.2.1. Photocatalytic NPs

First, 20 mL of a 0.1 mol/L AgNO_3_ standard solution was transferred to 10 mL of deionized water using a pipette and stirred thoroughly. Next, 0.073 g of HCl was dissolved in 10 mL of deionized water and added dropwise to the aforementioned solution. To obtain a milky white suspension, the solution was stirred with a magnetic stirrer at room temperature for 2 h and on a heated stirrer at 60 °C for 1 h. The thoroughly mixed suspension was centrifuged at a rate of 4000 r/min. Then, the precipitate was washed three times with water and ethanol. Afterwards, the sediment was transferred into a vacuum drying oven and heated to 60 °C for 12 h. After cooling, the dried precipitate was ground to obtain AgCl nanoparticles.

**Figure 1:**
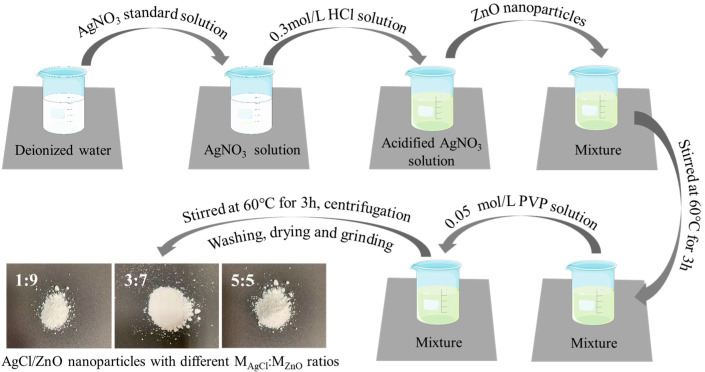
Schematic diagram of the process for preparing AgCl/ZnO nanoparticles.

Next, 0.1112 g of PVP was dissolved in 20 mL of anhydrous ethanol to form a 0.05 mol/L PVP solution as the cementing agent for ZnO and AgCl. Afterward, 30 mL of a 0.1 mol/L AgNO_3_ standard solution was transferred into a beaker that held 10 mL of deionized water. The solution was mixed thoroughly until it was clear and colorless. After that, 10 mL of a 0.3 mol/L HCl solution was slowly introduced into the beaker, and stirring continued with a magnetic stirrer for 2 h at room temperature. To the acidified AgNO_3_ aqueous solution, ZnO nanoparticles (0.243 g, 0.567 g, and 2.187 g) were added, and the solution was stirred at 60 °C for 3 h. Finally, 10 mL of a 0.05 mol/L PVP solution was added to the mixture and stirred continuously at 60 °C for 3 h. The thoroughly mixed suspension was centrifuged at a rate of 4000 r/min, and the precipitate was washed three times with water and ethanol. Afterwards, the sediment was transferred into a vacuum drying oven and heated to 60 °C for 12 h. After cooling, the dried precipitate was ground to obtain composite nanoparticles of AgCl/ZnO with different molar ratios (M_AgCl_:M_ZnO_ = 1:9, 3:7, and 5:5). A schematic diagram illustrating the process of preparation is shown in Figure 1.

#### 2.2.2. Nanofiber Membranes Supported by Photocatalytic NPs

**Figure 2:**
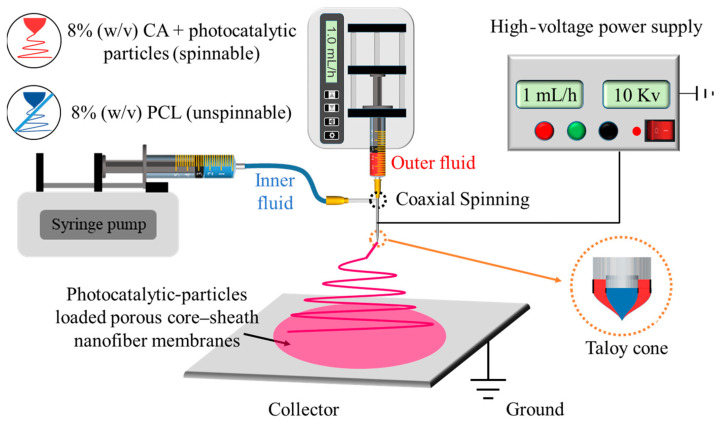
Schematic diagram of the preparation process of electrospun nanofibers.

Next, 0.8 g of CA was dissolved in 10 mL of HFIP, and 0.08 g of photocatalytic NPs were added to the solution. The mixture was stirred with a magnetic stirrer for 24 h until a uniform white suspension was obtained as the sheath fluid. Photocatalytic NPs of ZnO, AgCl, AgCl/ZnO (M_AgCl_:M_ZnO_ = 1:9), AgCl/ZnO (M_AgCl_:M_ZnO_ = 3:7), and AgCl/ZnO (M_AgCl_:M_ZnO_ = 5:5) were used to fabricate F1, F2, F3, F4, and F5, respectively. Then, 0.8 g of PCL was dissolved in 10 mL of CF and stirred with a magnetic stirrer for 12 h until a uniform, colorless, and transparent solution was obtained as the core fluid. The obtained sheath and core fluids were used to execute coaxial electrospinning. The collector distance was set to 15 cm, the flow rate of the core and sheath solutions was maintained at 1.0 mL/h, and the voltage was set to 10 kV, as shown in Figure 2.

### 2.3. Characterization

#### 2.3.1. Morphology and Structure

The surface morphology was examined under an accelerated voltage of 30 kV using a field emission scanning electron microscope (FESEM, Quanta FEG 450, FEI Corporation, USA). First, the sample stage was cleaned thoroughly, and a conductive adhesive was applied to its surface. The samples were placed on the conductive adhesive, and a Au coating was sprayed onto the surface using a coating instrument to provide conductivity to the samples. 

The photocatalytic particles and the fiber membrane loaded with photocatalytic particles were placed on a copper grid, and the internal structure was examined using a transmission electron microscope (TEM, Tecnai G2 F30, FEI Corporation, USA) at an accelerating voltage of 100 kV.

The specific surface area, pore volume, and pore size distribution of the electrospun nanofiber membranes were analyzed using a gas adsorption analyzer (ASAP2020, micromeritics, USA) providing nitrogen adsorption–desorption isotherms. The samples were heated and subjected to vacuum degassing to remove adsorbed impurities on their surfaces.

#### 2.3.2. Physical and Chemical State

The synthesized samples were characterized using an X-ray diffractometer (XRD, Karlsruhe, Germany) employing CuK α (λ = 1.541 Å) radiation as the X-ray source, with a scanning range of 10–70°.

X-ray photoelectron spectroscopy (XPS, ESCALAB Xi+, ThermoFischer, USA) was used to obtain XPS data.

The functional groups present in the coaxial electrospun nanofiber membranes were analyzed using Fourier transform infrared spectroscopy (FTIR, PerkinElmer, Billerica, MA, USA). KBr was ground together with the measured sample. The instrument’s scanning range was from 500 cm^−1^ to 4000 cm^−1^ with a resolution of 2 cm^−1^.

The UV absorption performance of the samples was characterized using a evaluate (Lambda 750, PerkinElmer, Billerica, USA). BaSO_4_ was pressed and treated as a background. Subsequently, a small amount of the photocatalyst was placed on its surface and pressed. The scan range was 300–800 nm.

#### 2.3.3. Photocatalytic Performance Testing

The photocatalytic performance of the samples was tested using a 300 W xenon lamp (PLS-SXE300D/300DUV, PerfectLight, China) to simulate sunlight. First, 10 mg of a sample was added to 100 mL of an MB solution and stirred for 30 min under dark conditions using a magnetic stirrer to achieve adsorption equilibrium between the functional particles and the organic pollutant solution. Second, the xenon lamp was positioned at a height of 15 cm above the MB solution. After turning on the xenon lamp, 3 mL of the solution was extracted and centrifuged every 10 min. Then, the absorbance of the solution was measured using a UV–visible spectrometer. The photodegradation rate of the organic pollutants was determined using the formula Q = (C_0_ − Ct)/C_0_ × 100%, where C_0_ and Ct represent the absorbance values of the solution at time *t* before and after irradiation with a xenon lamp, respectively, and Q is the degradation rate. After undergoing each photocatalytic degradation process, the sample was thoroughly rinsed with ethanol and deionized water multiple times and then dried at 60 °C for 24 h. Afterwards, the cyclic stability of the sample was examined. The error bar was calculated from three independent experiments.

#### 2.3.4. Analysis of Photocatalytic Mechanism

The photoluminescence (PL) spectrum of the functional fiber membrane was measured using an Edinburgh steady-state/transient fluorescence spectrometer (FLS1000, Edinburgh Instruments Ltd., UK) with an excitation wavelength of λ = 254 nm.

First, 5 mg of the photocatalyst-supported nanofiber membranes were crushed, ground, and dispersed in 1 mL of ethanol (containing 20 μL of Nafion) to prepare a slurry. The slurry was coated onto a fluorine-doped tin oxide transparent glass substrate and dried to create a working electrode. A carbon rod and a Ag/AgCl electrode were used as the counter electrode and the reference electrode, respectively. The photocatalyst’s photocurrent response (I-t) was measured under a switchable illumination cycle of 0.4 V and 100 s using a 300 W xenon lamp as a simulated solar light source. The distance between the photocatalyst and the xenon lamp was maintained at around 16 cm (with the xenon lamp operating at 14 A with an approximate light output power density of 200 mW∙cm^−2^). Electrochemical impedance spectroscopy and Mott–Schottky curves were measured under open-circuit conditions with a disturbance voltage of 5 mV in the frequency range of 0.01 Hz to 100 kHz.

Next, 1 mmol of Na_2_-EDTA, IPA, and P-BQ were separately added to the MB solution as free radical scavengers. During the photocatalytic process, Na_2_-EDTA, IPA, and P-BQ captured holes (h^+^), hydroxyl radicals (·OH), and superoxide radicals (·O_2_^−^), respectively. This condition allowed us to determine which type of free radical was the main factor affecting the photocatalytic degradation efficiency of MB.

## 3. Results and Discussion

### 3.1. Photocatalytic Nanoparticles

It is widely recognized that AgCl, ZnO, and their AgCl/ZnO complexes can be utilized as photocatalysts. The ZnO utilized in this article was commercially available, and AgCl and AgCl/ZnO were synthesized following the method described in Section 2.2.

**Figure 3:**
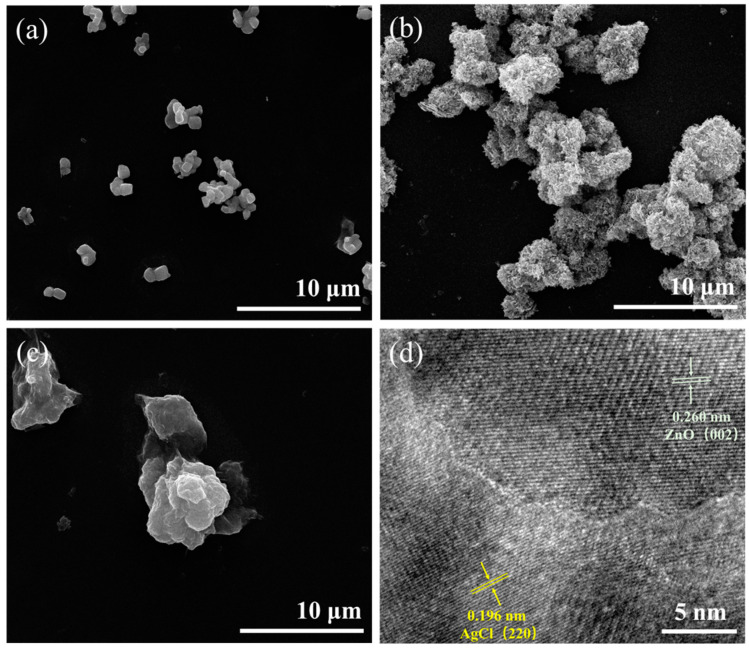
SEM images of (**a**) AgCl NPs, (**b**) ZnO NPs, and (**c**) AgCl/ZnO NPs (M_AgCl_: M_ZnO_ = 5:5) and (**d**) TEM image of AgCl/ZnO NPs (M_AgCl_: M_ZnO_ = 5:5).

The morphology and structure of these photocatalytic nanoparticles are shown in Figure 3. The prepared AgCl NPs exhibited a uniform size, an irregular cubic structure, and excellent dispersibility. The ZnO NPs had a size range of 20–30 nm and exhibited severe aggregation, which was due to the high surface activation energy or alterations in the surface charge state of the nanoparticles. The series of AgCl/ZnO NPs shared similar morphologies. As a representative of the series materials, an SEM image of AgCl/ZnO (M_AgCl_:M_ZnO_ = 5:5) is shown in Figure 3c. The surface morphology of the composite nanoparticles changed relative to individual AgCl and ZnO, and it effectively improved the agglomeration of ZnO. Considerable intermixing of the two types of particles was also observed, and a heterostructure was formed. The TEM image in Figure 3d provides further evidence and clearly shows the interface formed by ZnO and AgCl nanoparticles with lattice spacings of 0.260 and 0.196 nm, respectively [27].

**Figure 4:**
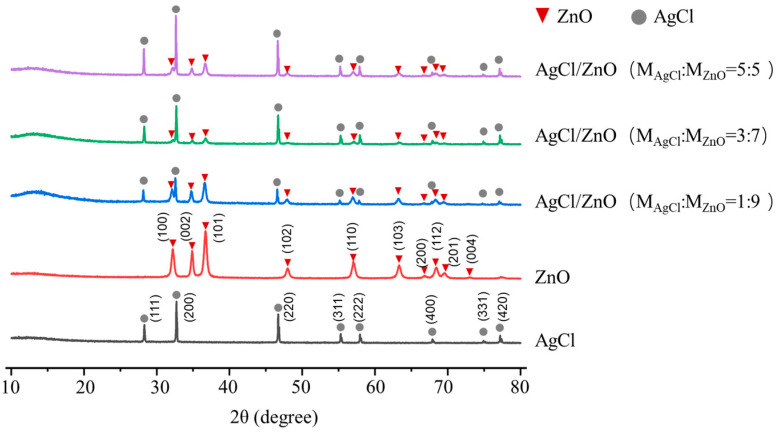
XRD patterns of ZnO NPs, AgCl NPs, and AgCl/ZnO NPs with different molar ratios.

Figure 4 presents the XRD patterns of the photocatalytic nanoparticles. The peaks labeled with red triangles “
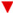
” and gray circles “
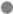
” match the standard data of ZnO (JCPDS card No. 36-1451) and AgCl (JCPDS card No. 31-1238), respectively [27]. The characteristic peaks of AgCl and ZnO can be identified in the series of AgCl/ZnO NPs, with no other crystallization peaks present, indicating the high purity of the AgCl/ZnO NPs. This is because the AgCl and AgCl/ZnO NPs in this work were prepared under dark conditions, so there was no reduction or a minimal reduction of AgCl. As shown in Supplementary Figure S1, the XRD spectrum of zinc oxide remained largely unchanged after the photocatalytic reaction. However, a crystalline peak with a position at 38.8, which corresponds to the standard data of silver (JCPDS card No. 04-0783), was identified within the AgCl and AgCl/ZnO NPs after the photocatalytic reaction. These data demonstrate the decomposition of silver chloride during the photocatalytic process.

**Figure 5:**
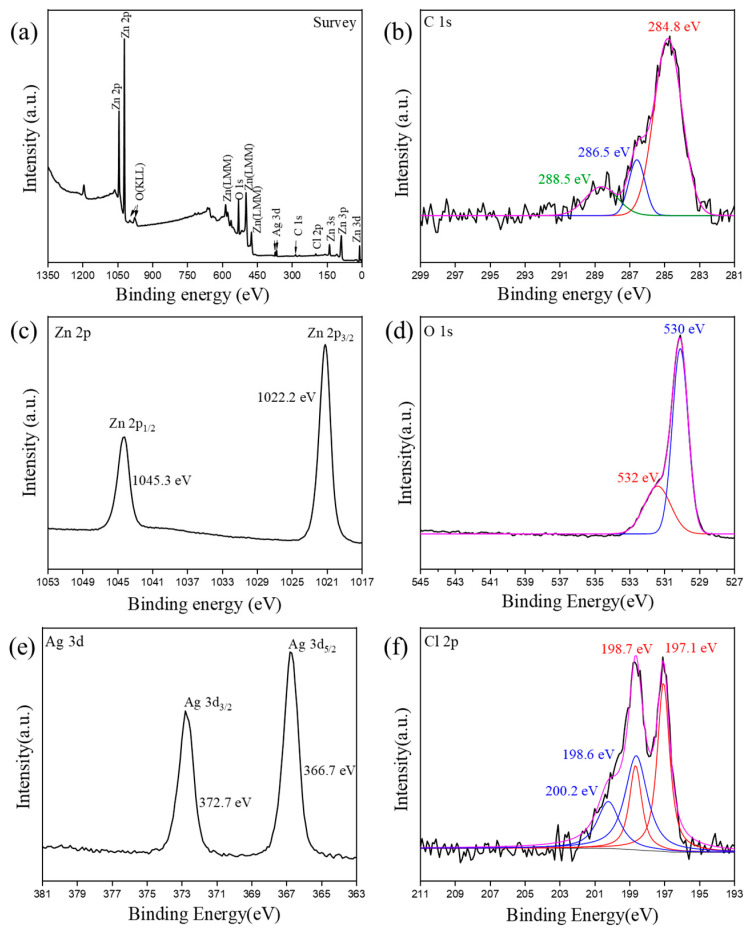
XPS spectrum of AgCl/ZnO NPs (MAgCl:MZnO = 5:5): (**a**) survey, (**b**) C *1s*, (**c**) Zn *2p*, (**d**) O *1s*, (**e**) Ag *3d*, and (**f**) Cl *2p*.

Furthermore, the details of the material composition and element valance state of AgCl/ZnO NPs (MAgCl: MZnO = 5:5) were analyzed using energy dispersive X-ray photoelectron spectroscopy (XPS). As indicated in the wide scan survey spectra (Figure 5a), the characteristic peaks of Zn *2p*, O *1s*, Ag *3d*, C *1s*, and Cl *2p* are observed. In Figure 5b, the C 1 s spectrum consists of deconvoluted peaks at approximately 288.5 eV, 286.5 eV, and 284.8 eV, which are signatures of C = O, C-N, and C-C carbon species, respectively [28]. In the magnified XPS spectrum of Zn *2p* (Figure 5c), the two spin orbital peaks at 1045.3 eV and 1022.2 eV belong to Zn *2p* 1/2 and Zn *2p* 3/2, respectively, reflecting the stable Zn^2+^ state [29]. The O 1 s spectrum shown in Figure 5d reveals oxygen components situated at around 530 eV, and 532 eV, which are typically related to ZnO, and C = O bonds, respectively [29]. In the Ag 3d spectrum in Figure 5e, the clear peaks at 372.7 eV and 366.7 eV are associated with two satellite peaks corresponding to Ag *3d* 3/2 and Ag *3d* 5/2, which is ascribed to the Ag in AgCl [30]. Shown in Figure 5f are peaks at 198.7 eV and 197.1 eV, which are signatures of the Cl^−^ in AgCl, and peaks at 200.2 eV and 198.6 eV, which are signatures of the Cl^0^ [15,30].

**Figure 6:**
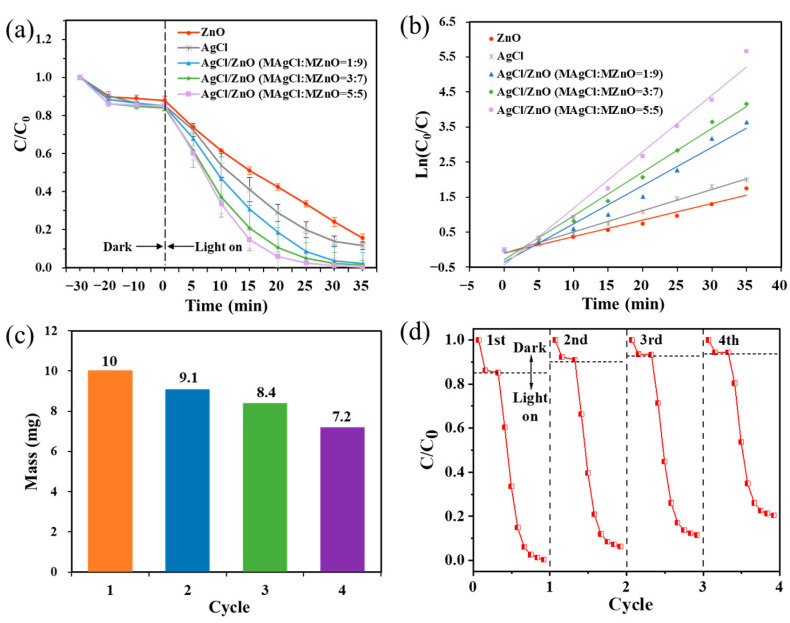
(**a**) Photodegradation activity and (**b**) corresponding first-order kinetic fitting curves of different photocatalytic NPs toward MB under simulated solar light irradiation. (**c**) Mass and (**d**) photocatalytic performance of AgCl/ZnO (M_AgCl_:M_ZnO_ = 5:5) after each cycle.

The operation of the photocatalytic experiment is detailed in Section 2.3.3. As shown in Figure 6a, the photocatalytic efficiency of AgCl/ZnO NPs was much higher than those of the individual AgCl NPs and ZnO NPs. As the content of AgCl increased, so too did the photocatalytic efficiency of the AgCl/ZnO NPs. The kinetic analysis of photocatalytic MB degradation is illustrated in Figure 6b. The fitting correlation coefficients are 0.96717, 0.99187, 0.96742, 0.98773, and 0.98041, respectively, indicating that the photocatalytic processes of MB degradation by the five different photocatalysts all follow the first-order kinetic model. The lower photocatalytic efficiency of AgCl and ZnO may be due to the easier recombination of electrons in the valence band (VB) with defects (holes) in the conduction band (CB). The formation of a heterojunction between AgCl and ZnO can effectively suppress the recombination of electrons and holes. O_2_ can be reduced to ·O_2_^-^ by the electrons on the CB, whereas H_2_O can be oxidized to ·OH by the holes in the VB. When the molar ratio of AgCl to ZnO is 1:1, the heterojunction between AgCl and ZnO becomes highly enriched, effectively suppressing electron–hole recombination and attaining the utmost photocatalytic efficiency.

Figure 6c shows that after four photocatalytic experiments, the mass of the photocatalyst decreased from 10 mg to 7.2 mg. Figure 6d indicates that as the cyclic experiments progressed, the photocatalytic degradation efficiency of MB decreased. The degradation rates for the first, second, third, and fourth cycles were 99.70%, 93.76%, 88.56%, and 79.64%, respectively. This was mainly due to the loss of photocatalytic particles during the centrifugation and drying processes of the photocatalytic experiments.

### 3.2. Loading Photocatalytic NPs Onto Electrospun Nanofibers

In order to improve the recycling efficiency of the photocatalysts, the aforementioned photocatalytic particles were loaded onto nanofiber membranes via multifluid electrospinning. With PCL as the core layer in the coaxial electrospinning process, fibers with improved mechanical properties were obtained to compensate for the weakened mechanical performance of the porous fibers.

**Figure 7:**
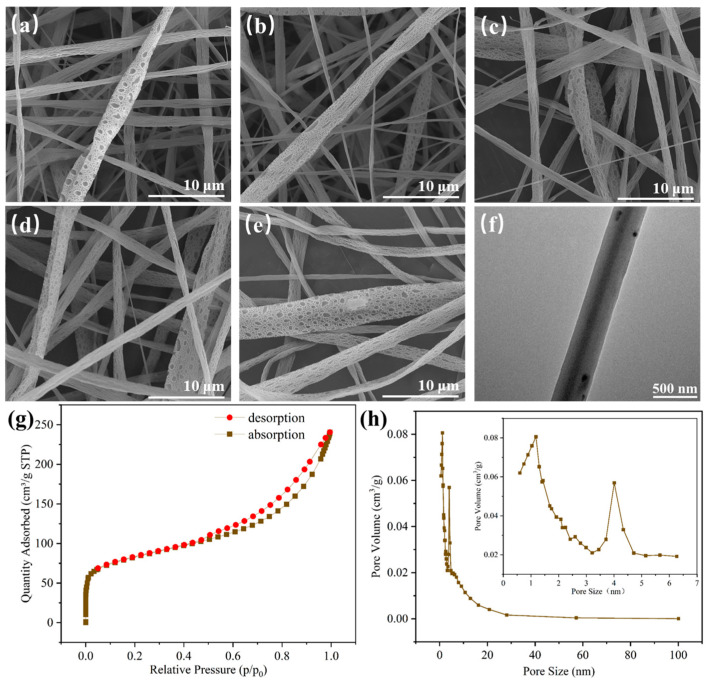
(**a**–**e**) SEM images of F1–F5, (**f**) TEM image, (**g**) nitrogen adsorption–desorption isotherm, and (**h**) pore size distribution curve of F5.

As shown in Figure 7a–e, SEM observations revealed that there were numerous irregular pores and a minor quantity of photocatalytic particles on the surfaces of electrospun nanofibers F1–F5. The presence of these irregular pores can be attributed to the rapid evaporation of HFIP in the sheath solution during electrospinning, resulting in a decrease in the surface temperature of the fibers. Consequently, the surrounding water molecules undergo condensation and form water droplets attached to the surfaces of nanofibers. Additionally, the core solvent, CF, is volatile and diffuses to the surfaces of nanofibers as HFIP evaporates. CF is sparingly soluble in water, resulting in the formation of irregular structures when CF comes into contact with water. Once water droplets and CF completely evaporate, irregular pores are left behind on the surfaces of nanofibers. The successful loading of the photocatalytic nanoparticles and the core–sheath structure of the fibers could be further verified through TEM analysis, as shown in Figure 7f.

The nitrogen adsorption–desorption isotherm of F5, which corresponds to a type II isotherm [31] that is suitable for mesoporous materials, is shown in Figure 7g. The specific surface area of F5 was calculated to be 293.54 m^2^/g. The pore size distribution curve shown in Figure 7h revealed that the pores on F5 mainly consisted of micropores, mesopores, and a small number of macropores, and the average pore size was 6.22 nm.

**Figure 8:**
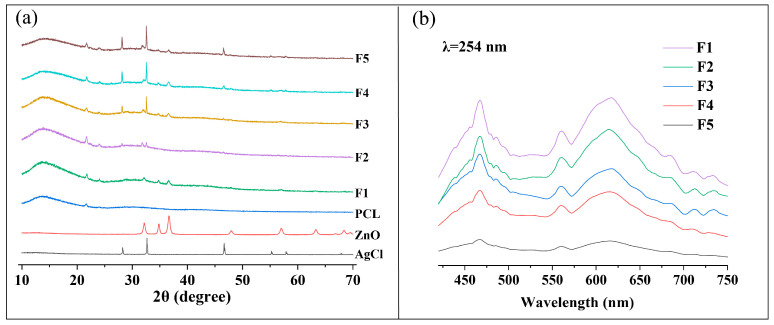
(**a**) XRD patterns of PCL, AgCl NPs, ZnO NPs, and nanofiber membranes F1–F5 and (**b**) photoluminescence spectra of nanofiber membranes F1–F5.

Figure 8a shows the XRD patterns of PCL, AgCl NPs, ZnO NPs, and coaxial electrospun nanofiber membranes F1–F5. PCL had two diffraction peaks at around 21.7° and 24.0°, indicating its semicrystalline nature. PCL is composed of two structural forms, namely, ordered crystalline and disordered amorphous regions. The polymerization of PCL involves the ring-opening polymerization of ε-caprolactone, resulting in crystalline regions in the middle of the polymer chains and amorphous regions surrounding them. Therefore, PCL is considered a semicrystalline polymer. The peaks at 2θ = 31.8°, 34.4°, 36.3°, 47.5°, and 56.6° observed in the XRD patterns of the F1 and F3–F5 nanofiber membranes correspond to the (100), (002), (101), (102), and (110) crystal planes of ZnO, respectively, indicating the successful loading of ZnO NPs onto the surfaces of the nanofibers. The peaks at 2θ = 27.8°, 32.2°, 46.2°, 54.8°, and 57.5° observed in the XRD patterns of the F2 and F3–F5 nanofiber membranes correspond to the (111), (200), (220), (311), and (222) crystal planes of AgCl, respectively, indicating the successful loading of AgCl NPs onto the surfaces of the nanofibers. With increasing AgCl contents, the diffraction peaks of AgCl in F3–F5 gradually increased, whereas the diffraction peaks of ZnO gradually decreased.

PL is used to study the transfer and separation of photo-induced charge carriers in electrospun nanofiber membranes [32]. Typically, the lower the peak intensity of PL, the lower the recombination rate of photo-induced charge carriers and the higher the photocatalytic activity. As shown in Figure 8b, F1 and F2 exhibited higher peak PL intensities than F3–F5, and the peak PL intensities of F3–F5 decreased because the number of AgCl/ZnO heterojunctions increased. This result was consistent with the photocatalytic MB degradation efficiency of unloaded photocatalytic particles.

**Figure 9:**
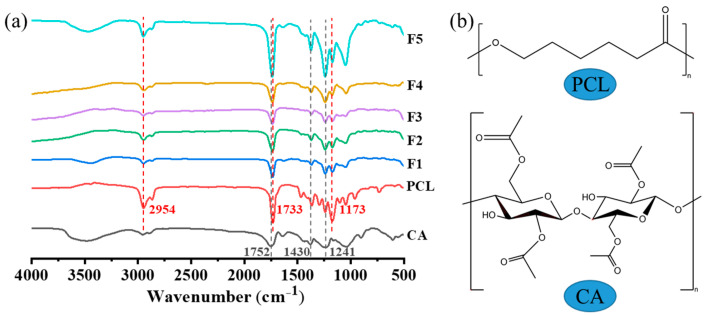
(**a**) FTIR spectra of the raw materials PCL and CA, and nanofiber membranes F1–F5. (**b**) Molecular structures of PCL and CA.

Analyses and characterizations of the functional groups in the polymer substrates and coaxial electrospun nanofiber membranes were performed, as shown in Figure 9a. In the infrared spectrum of CA, the peak at 1752 cm^−1^ corresponds to the C=O stretching vibration of the carbonyl group, whereas the peaks at 1241 cm^−1^ and 1430 cm^−1^ correspond to C-O tensile vibration and C-H tensile vibration, respectively [33]. In the infrared spectrum of PCL, the peaks of C-H, C=O, and C=O stretching vibrations appear at 2954 cm^−1^, 1733 cm^−1^, and 1173 cm^−1^, respectively [34]. After loading the photocatalytic particles onto the nanofibers, a noticeable shift in the C=O vibration peak toward lower wave numbers was observed, which suggested the formation of hydrogen bonds between CA and the photocatalytic particles.

### 3.3. Photocatalytic Performance of the Nanofiber Membranes

**Figure 10:**
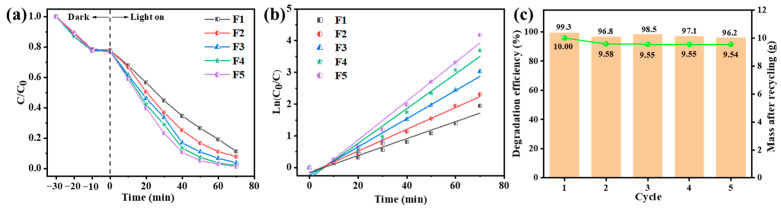
(**a**) Photodegradation activity, (**b**) corresponding first-order kinetic fitting curves of MB under simulated solar light irradiation of F1–F5, and (**c**) cyclic experiment on F5.

Photocatalyst-loaded porous nanofiber membranes F1–F5 were added to a 100 mL 10 mg/L MB solution. As shown in Figure 10a, after 30 min of dark treatment, the adsorption and desorption of MB on the porous nanofiber membranes reached equilibrium, and the absorption of MB was greater than when using photocatalytic nanoparticles. Subsequently, after simulated solar light irradiation for 70 min, the adsorption degradation rates of MB on F1–F5 were 85.67%, 90.07%, 95.18%, 97.50%, and 98.47%, respectively. The correlation coefficients of the kinetic fitting were 0.96428, 0.98785, 0.98032, 0.97579, and 0.97996, as shown in Figure 10b, indicating compliance with the first-order kinetic model. In addition, a five-cycle repeated experiment was conducted on F5. The cyclic degradation efficiency and the remaining mass of the membrane were recorded after each cycle. As presented on the left y-axis in Figure 10c, even after five cycles, F5 maintained a photocatalytic efficiency of over 95% with only minor variations. This result suggests that the photocatalyst was firmly fixed on the porous nanofiber membranes and formed a stable structure that maintained good dispersion and catalytic activity even after multiple cycles. The nanofiber membrane possessed a porous structure and a large surface area, allowing the photocatalyst to be exposed on the surface and increasing the area of contact with MB. Additionally, the pore structure could scatter light effectively within the fiber membrane, thereby enhancing the photocatalytic reaction. As shown in Figure S2, the structure of the nanofiber membrane remained after the photocatalytic reaction.

### 3.4. Photocatalytic Mechanism

**Figure 11:**
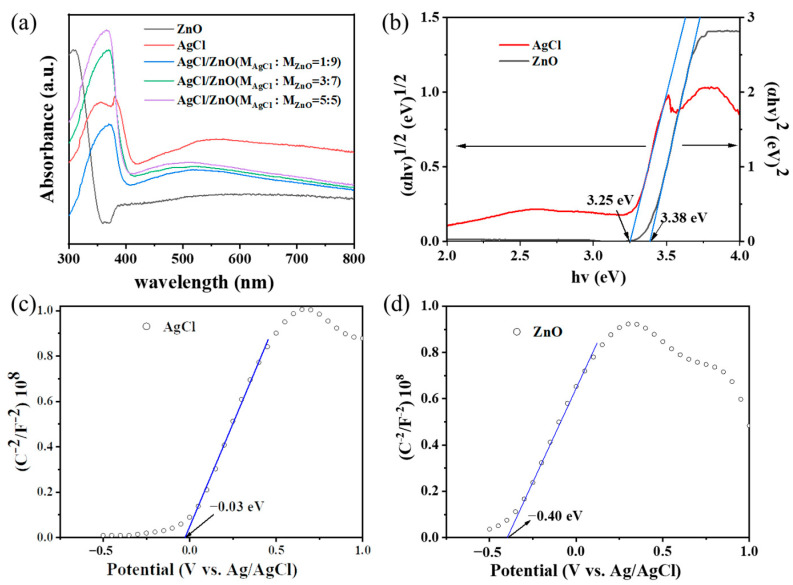
(**a**) UV–visible absorption spectra of AgCl NPs, ZnO NPs, and AgCl/ZnO NPs. (**b**) Tauc curves of ZnO NPs and AgCl NPs. Mott–Schottky curves of (**c**) ZnO NPs and (**d**) AgCl NPs.

To gain a deeper insight into the photocatalytic mechanism and band structure of AgCl/ZnO heterojunctions, the photocatalytic nanoparticles were first characterized using UV–Vis absorption spectra, as shown in Figure 11a. The absorption spectrum of pure ZnO exhibited strong absorption below 400 nm, indicating the efficient absorption of UV light due to the charge transfer from the VB (O 2p orbitals) to the CB (Zn 4s orbitals). In comparison with ZnO, the absorption spectra of AgCl NPs and AgCl/ZnO NPs showed strong absorption below 400 nm and between 400 and 800 nm. The bandgap widths of ZnO and AgCl could be calculated using the Kubelka–Munk equation (αhv) = A(hv − E_g_)^n/2^, where the integer *n* values of the direct semiconductor (ZnO) and the indirect semiconductor (AgCl) were 1 and 4, respectively [35]. As shown in Figure 11b, the bandgap (E_g_) values of AgCl and ZnO were 3.25 and 3.38 eV, respectively. The Mott–Schottky [36] curves of AgCl and ZnO, shown in Figure 11c,d, suggest that AgCl and ZnO are n-type semiconductors. The flat-band potentials (E_FB_) of AgCl and ZnO relative to Ag/AgCl at pH 7 were −0.03 and −0.40 V, respectively. After conversion to the normal hydrogen electrode (NHE), the E_CB_ values were calculated to be −0.033 and −0.403 V (E_CB_(NHE) = E_CB_(Ag/AgCl) + 0.197). Therefore, the corresponding valence (E_VB_ = E_g_ + E_CB_) values of AgCl and ZnO were 3.217 and 2.977 eV, respectively.

**Figure 12:**
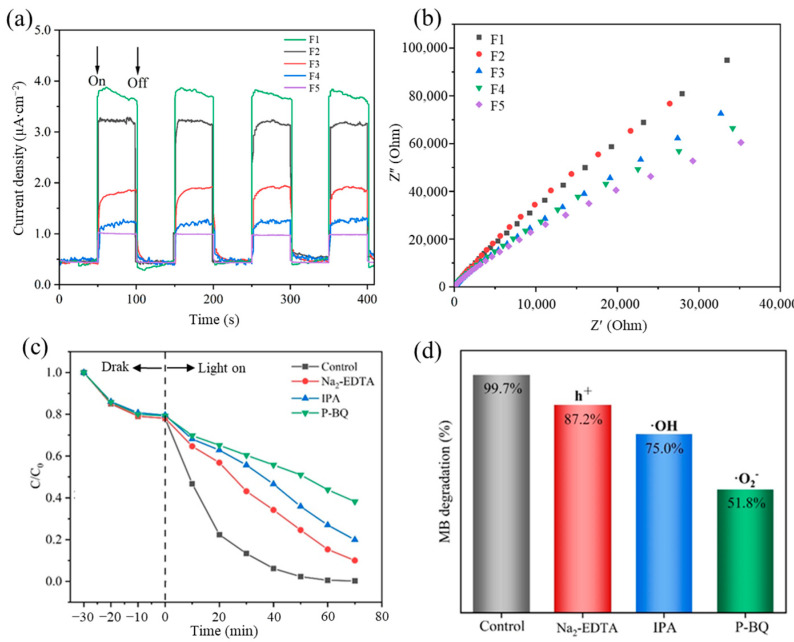
(**a**) Transient photocurrent responses of F1–F5 under simulated solar light irradiation. (**b**) Electrochemical impedance spectra of F1–F5. (**c**) Free radical trapping experiment on F5 under simulated solar light irradiation. (**d**) Degradation efficiency of MB after the addition of free radicals.

The I-t testing [37] of F1–F5 was performed in a 0.2 M Na_2_SO_4_ electrolyte solution to further demonstrate the effective separation of photogenerated charge carriers in the nanofiber membranes. As shown in Figure 12a, when the xenon lamp was turned on to illuminate the sample, the electrons on the sample surface were excited. The concentration of charge carriers on the sample surface increased, resulting in a linear increase in the current density, and a stable state was reached at a certain amplitude. When the xenon lamp was turned off, the number of excited electrons on the sample surface decreased, and the concentration of charge carriers decreased accordingly, leading to a decrease in the current density and a continued stable state. With 100 s as one switching cycle, the peak intensity of the current density when the xenon lamp was turned on and off remained relatively constant in each cycle. The photocurrent densities of F3–F5 were much higher than those of F1 and F2, revealing the strong light absorption capability of the AgCl/ZnO nanoparticles, which could promote the separation of photogenerated charge carriers. F5 exhibited the highest photoresponse activity, which was consistent with the PL results.

The electrochemical impedance spectra were measured in a 0.2 M Na_2_SO_4_ electrolyte solution to further demonstrate the effective separation of photogenerated charge carriers in F1–F5 [38]. The size of the electrochemical reaction region on an electrode surface, which is often referred to as the electrode polarization layer radius or effective diffusion layer radius, reflects the size of the electrochemical reaction region. Generally, a small electrochemical reaction region on an electrode surface indicates a fast diffusion of reactants, which helps improve the efficiency of electron–hole separation. Figure 12b shows that the radii of F3–F5 were smaller than those of F1 and F2, and as the molar ratio of AgCl to ZnO increased, the radii gradually decreased. F5 exhibited high efficiency in the separation of electrons and holes, indicating that the formed heterojunctions suppressed the recombination of photogenerated carriers to a great extent. This result further confirmed the presence of a larger number of electrons and holes participating in the photocatalytic reaction in F5.

To investigate the influence of free radicals on MB degradation during the photocatalytic process, this study introduced 0.1 mmol Na_2_-EDTA, P-BQ, and IPA as free radical scavengers into the F5 system for MB degradation; these scavengers can capture h^+^, ·O_2_^−^, and ∙OH free radicals, respectively. As shown in Figure 12c,d, the addition of P-BQ considerably reduced the degradation efficiency of MB. This finding indicates that ·O_2_^−^ plays a dominant role as the key free radical in the degradation process of MB. P-BQ reacts with and neutralizes ·O_2_^−^ free radicals, thereby reducing the amount of ·O_2_^-^ in the photocatalytic reaction and consequently lowering the rate and efficiency of the photocatalytic reaction [39]. Moreover, P-BQ can be competitively adsorbed onto the photocatalysts, obstructing the adsorption of organic molecules and impeding the progress of the photocatalytic reaction, which also leads to a decrease in photocatalytic efficiency. The addition of Na_2_-EDTA and IPA also resulted in a certain degree of photocatalytic efficiency reduction, indicating the participation of h^+^ and ∙OH. Therefore, h^+^, ∙OH, and ·O_2_^−^ all play significant roles in the photocatalytic decomposition process.

**Figure 13:**
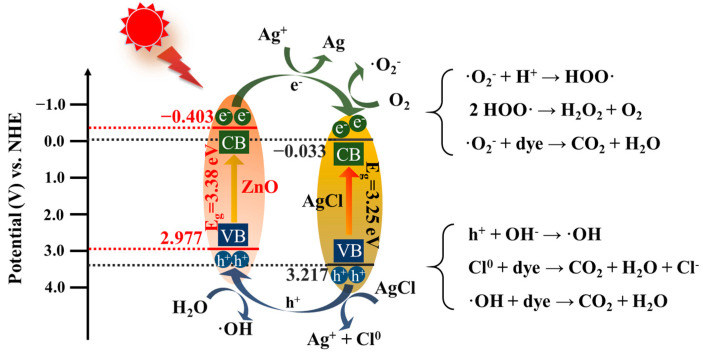
Possible photocatalytic mechanism of the degradation.

The photocatalyst was in an excited state under simulated solar light irradiation. The electrons (e^−^) in AgCl and ZnO underwent a transition from the VB to the CB, leaving corresponding holes in the VB. At this point, the CB potential of AgCl was more positive than that of ZnO, so electrons from the CB of ZnO transitioned to the CB of AgCl and gradually accumulated. Based on the above analysis, a photocatalytic mechanism was proposed in Figure 13. Firstly, under UV light irradiation, photogenerated electron–hole pairs (e^−^–h^+^) were formed in ZnO NPs. AgCl decomposes under light to produce Ag, which absorbed UV light, and generated Ag^+^ and e^−^. Secondly, some e^−^ from the CB reacted with Ag^+^ to form Ag and the other e^−^ were expected to be trapped by O_2_ in solution to generate ·O_2_^−^ and other oxygen species like HOO· and H_2_O_2_. The generated radicals can react with dyes [40]. Thirdly, the h^+^ could react with AgCl to form Ag^+^ and Cl^0^, and a part of h^+^ generated by ZnO reacted with H_2_O to form ·OH. The Cl^0^ and ·OH could also react with the dyes. Through the synergistic action of ·O_2_^−^, ·OH, h^+^ and Cl^0^, the MB were degraded into CO_2_ and H_2_O.

## 4. Conclusions

Water pollution caused by organic dyes necessitates more efficient and sustainable treatment strategies. This study demonstrated a one-step method for fabricating porous nanofiber membranes loaded with functional particles that had large surface areas and high photocatalytic efficiency for MB. With a stable core layer in the coaxial electrospinning process, nanofibers with improved mechanical properties were obtained to compensate for the weakened mechanical performance of the porous fibers. A porous nanofiber membrane loaded with AgCl/ZnO (M_AgCl_:M_ZnO_ = 5:5) showed the highest photodegradation efficiency for MB, and even after five cycles of repeated experiments, the mass and photocatalytic efficiency of the nanofiber membrane remained above 95%, which successfully addressed the issues of photocatalytic efficiency loss during the photodegradation with AgCl/ZnO NPs. However, the porous structure only existed on the surfaces of the nanofibers, leaving room for further improvement in the structural design. Additionally, when loading functional particles onto the surfaces of porous nanofibers, attention should be paid to potential issues, such as particle detachment, which could affect photocatalytic efficiency. Although this study focused on the photocatalytic degradation of organic dyes, the porous nanofiber membrane could also be applied to address environmental issues such as heavy metal ion adsorption.

## Data Availability

The raw data supporting the conclusions of this article will be made available by the authors on request.

## Supplementary Materials

The following supporting information can be downloaded at: https://www.mdpi.com/article/10.3390/polym16060754/s1, Figure S1: XRD patterns of ZnO NPs, AgCl NPs, and AgCl/ZnO NPs with different molar ratios after photocatalytic reaction. Figure S2: SEM images of F1, F2, F3, F4, and F5 before (a, b, c, d, e) and after (a1, b1, c1, d1, e1) photocatalytic reaction. Scale bar: 500 nm.
