# Supplementary material for: Coaxial Electrospun Porous Core–Shell Nanofibrous Membranes for Photodegradation of Organic Dyes
Source: Polymers (Basel). 2024 Mar 9;16(6):754. doi: 10.3390/polym16060754 (PMC10975244; doi:10.3390/polym16060754)
Supplement: Supplementary file 1 [file polymers-16-00754-s001.zip › polymers-2837976-supplementary.pdf]

## Supporting information

### Coaxial electrospun porous core–shell nanofibrous membranes for photodegradation of organic dyes

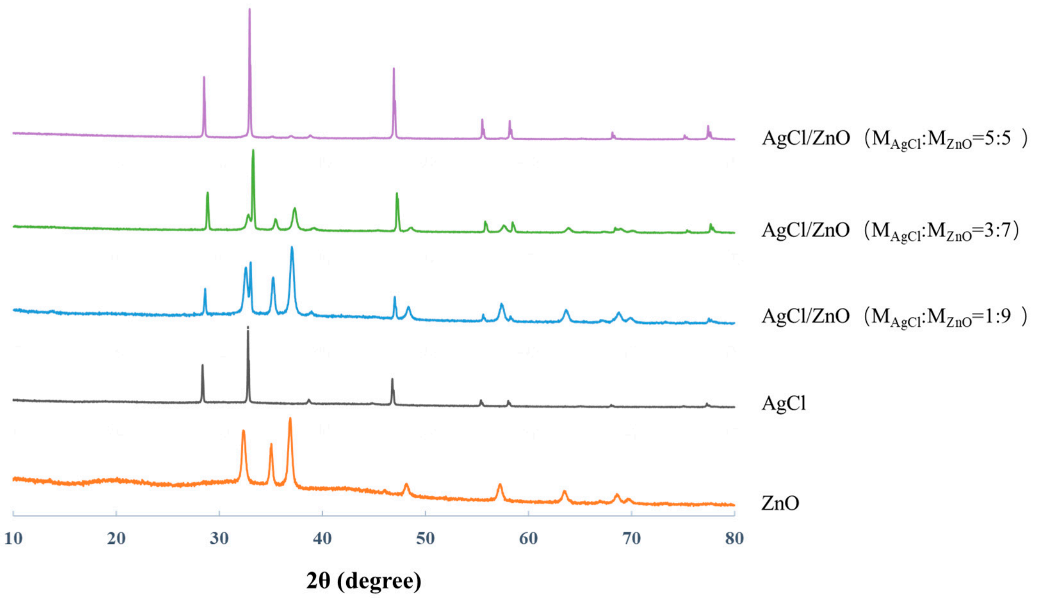


**Figure S1.** XRD patterns of ZnO NPs, AgCl NPs, and AgCl/ZnO NPs with different molar ratios after photocatalytic reaction.

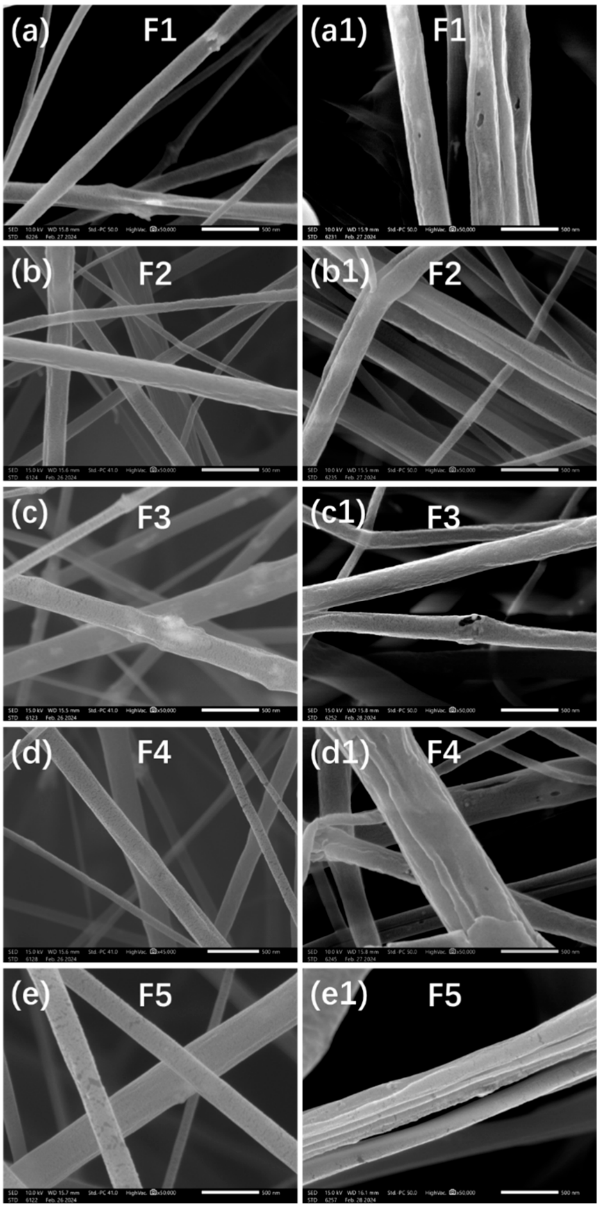


**Figure S2.** SEM images of F1, F2, F3, F4, and F5 before (a, b, c, d, e) and after (a1, b1, c1, d1, e1) photocatalytic reaction. Scale bar: 500nm.

The eletrospun nanofibers in the Figure S2 were prepared using a smaller spinneret than the one use for the nanofibers in the main manuscript, resulting in a smaller diameter of nanofibers and a certain difference in the pore structure. However, there was no significant changes in the morphology of the nanofibers before and after the photocatalytic reaction.
